# Immunomodulatory roles of metalloproteinases in rheumatoid arthritis

**DOI:** 10.3389/fphar.2023.1285455

**Published:** 2023-11-15

**Authors:** Yanqin Bian, Zheng Xiang, Yaofeng Wang, Qing Ren, Guoming Chen, Bei Xiang, Jianye Wang, Chengbo Zhang, Shaoqiang Pei, Shicheng Guo, Lianbo Xiao

**Affiliations:** ^1^ Institute of Arthritis Research in Integrative Medicine, Shanghai Academy of Traditional Chinese Medicine, Shanghai, China; ^2^ Guanghua Hospital Affiliated to Shanghai University of Traditional Chinese Medicine, Shanghai, China; ^3^ Li Ka Shing Faculty of Medicine, The University of Hong Kong, Hong Kong, Hong Kong SAR, China; ^4^ Department of Medical Genetics, School of Medicine and Public Health, University of Wisconsin-Madison, Madison, WI, United States

**Keywords:** matrix metalloproteinases, rheumatoid arthritis, bone destruction, angiogenesis, target

## Abstract

Rheumatoid arthritis (RA) is a chronic, autoimmune pathology characterized by persistent synovial inflammation and gradually advancing bone destruction. Matrix metalloproteinases (MMPs), as a family of zinc-containing enzymes, have been found to play an important role in degradation and remodeling of extracellular matrix (ECM). MMPs participate in processes of cell proliferation, migration, inflammation, and cell metabolism. A growing number of persons have paid attention to their function in inflammatory and immune diseases. In this review, the details of regulation of MMPs expression and its expression in RA are summarized. The role of MMPs in ECM remodeling, angiogenesis, oxidative and nitrosative stress, cell migration and invasion, cytokine and chemokine production, PANoptosis and bone destruction in RA disease are discussed. Additionally, the review summarizes clinical trials targeting MMPs in inflammatory disease and discusses the potential of MMP inhibition in the therapeutic context of RA. MMPs may serve as biomarkers for drug response, pathology stratification, and precision medicine to improve clinical management of rheumatoid arthritis.

## 1 Introduction

Rheumatoid arthritis (RA) is an autoimmune disorder characterized by persistent and chronic synovial inflammation that causes joint swelling, pain, and stiffness bilaterally leading to disability. The global prevalence of RA is approximately 0.4%–1.3% (Lin et al., 2020). RA involves a complex interplay among genotype, environmental triggers, and chance such as specific viruses and bacteria, promoting loss of tolerance to self-proteins that contain a citrulline residue and resulting in occurrence of RA. Currently, a range of treatment options including conventional and biologic antirheumatic drugs are available (Lin et al., 2020). However, none of them can be considered universally effective (Sokka et al., 1999; Aletaha and Smolen, 2018; Lin et al., 2020). Understanding of mechanisms of RA and the targeted development of new therapies is still a priority.

Matrix metalloproteinases (MMPs) are zinc-dependent endopeptidases belonging to the superfamily of metzincin proteases. They are produced by multiple cells and tissues to involve in the extracellular matrix (ECM) degradation and remodeling. MMPs play an important role in the development of RA disease. They can degrade ECM to destroy the integrity of synovial, cartilage and bone tissue. As a processing enzymes, MMPs have the ability to selectively cleave many non-matrix components present in the extracellular environment such as cell surface receptor, cytokines, chemokines, cell-cell adhesion molecules, clotting factors and other proteinases like binding proteins to involve in inflammation and immunity in RA (4, 5). Additionally, MMPs could directly activate signaling molecules, tumour necrosis factor (TNF), contributing to various aspects of immunity in RA disease (Khokha et al., 2013). The role of MMPs in immune cell development, function, and inflammatory response has drawn more attention to develop MMPs-target therapy in RA disease (Khokha et al., 2013; Cabral-Pacheco et al., 2020). This review will focus on the functions and mechanisms of MMPs in RA pathogenesis and the potential of targeting MMPs as a new therapeutic strategy in the clinical management of RA.

## 2 Regulation of MMPs expression

**FIGURE 1:**
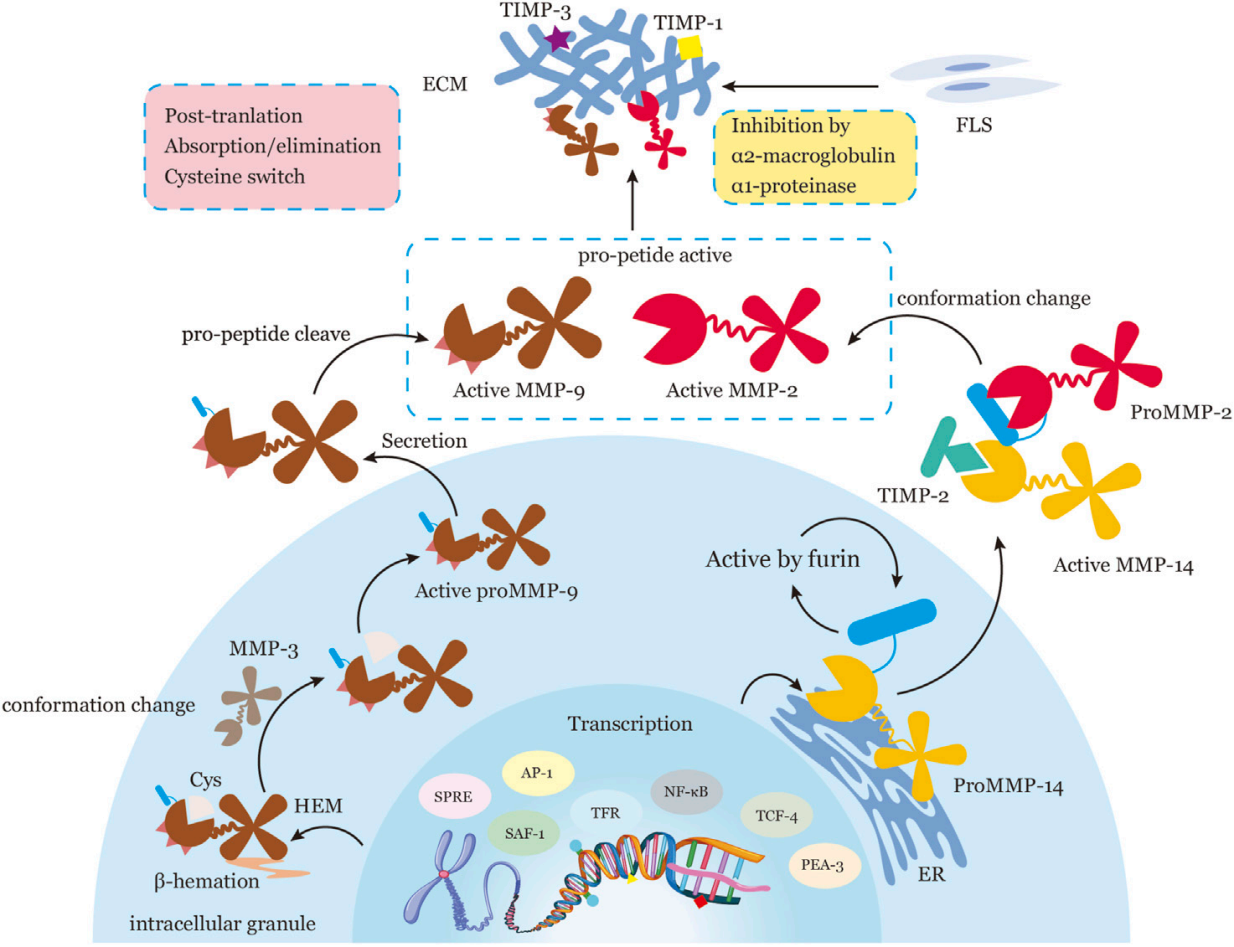
Representation of the Regulatory Processes of MMPs in Transcription, Activation of Zymogen Precursors, Interaction with Specific Components of the ECM, Inhibition by Endogenous Inhibitors, and Post-Translation. MMPs are subject to tight regulation at five levels: transcription, activation of zymogen precursors, interaction with specific components of the ECM, inhibition by endogenous inhibitors, and post-translational modification. During transcription, MMP expression is regulated by a variety of transcription factors, such as AP-1, PEA-3, SAF-1, NF-κB, SPRE, TRF, TCF-4, and epigenetic mechanisms, such as DNA methylation and/or chromatin remodeling with histone acetylation. MMPs are translated into inactive pro-forms or zymogens with a pro-domain that must be removed for activation. Pro-MMPs are activated in two ways: conformation change within the pro-peptide (e.g., proMMP-14 activation by furin in the normal secretory pathway intracellularly; pro-MMP2 activation through the formation of an MMP14-TIMP2-pro-MMP2 trimolecular complex at the cell surface) or intrinsic allostery of the MMP molecule (e.g., proMMP9 activation by β-hematin-induced truncation of a cysteine sulfhydryl group or Cys, processed by MMP3). Active MMPs interact with specific substrates to degrade the ECM. Activation of MMPs is controlled by specific binding with tissue inhibitors of metalloproteinases (TIMPs) and by non-specific proteinase inhibitors such as α1-proteinase inhibitor and α2-macroglobulin. Post-translational regulation of MMPs involves absorption and/or elimination of active proteases from the extracellular milieu. The precise mechanisms of post-translation regulation of MMP activity remain unclear. Note: Activator protein, AP-1; polyoma enhancer A binding protein-3, PEA-3; serum amyloid A-activating factor-1, SAF-1; surface protein releasing enzyme, SPRE; thyrotropin releasing factor, TRF; T cell factor-4, TCF-4; cysteine sulfhydryl group, Cys.

MMPs expression requires an extracellular stimulation for induction and activation via cytokines, chemokines, growth factors, or alterations in cell-matrix and cell-cell interactions (Cui et al., 2017; Laronha and Caldeira, 2020; de Almeida et al., 2022). As depicted in Figure 1, the expression of MMPs is tightly regulated via transcription, activation of zymogen precursors, interaction with specific components of the extracellular matrix (ECM), inhibition by endogenous inhibitors, and post-translational modifications (Galis and Khatri, 2002; Kessenbrock et al., 2010; Loffek et al., 2011). Transcriptional control of MMPs is involved in DNA methylation and chromatin remodeling with histone acetylation (Loffek et al., 2011; He et al., 2020). Studies have revealed that hypomethylation of MMP promoters can lead to increased enzyme expression in inflammatory disorders, such as MMP3, MMP9, and MMP13 in osteoarthritis chondrocytes (Roach et al., 2005), and MMP1 in RA synovial fibroblasts (Gaur et al., 2016). Activation of MMPs is regulated by specific binding with TIMPs and non-specific proteinase inhibitors, such as α1-proteinase inhibitor and α2-macroglobulin (Cui et al., 2017; Laronha and Caldeira, 2020). Post-translational modifications of MMPs are involved in absorption and/or elimination of active proteases from the environment (Cabral-Pacheco et al., 2020). Reactive oxygen and nitrogen species, proteinases, and organomercurials may disrupt the bond between the conserved cysteine sulfhydryl group and the active site zinc ion, a process known as “cysteine switch” mechanism of pro-MMP activation (Van Wart and Birkedal-Hansen, 1990). However, the mechanism underlying MMP expression at the post-translational level remains largely unclear and requires further investigation.

## 3 MMPs expression in RA

**FIGURE 2:**
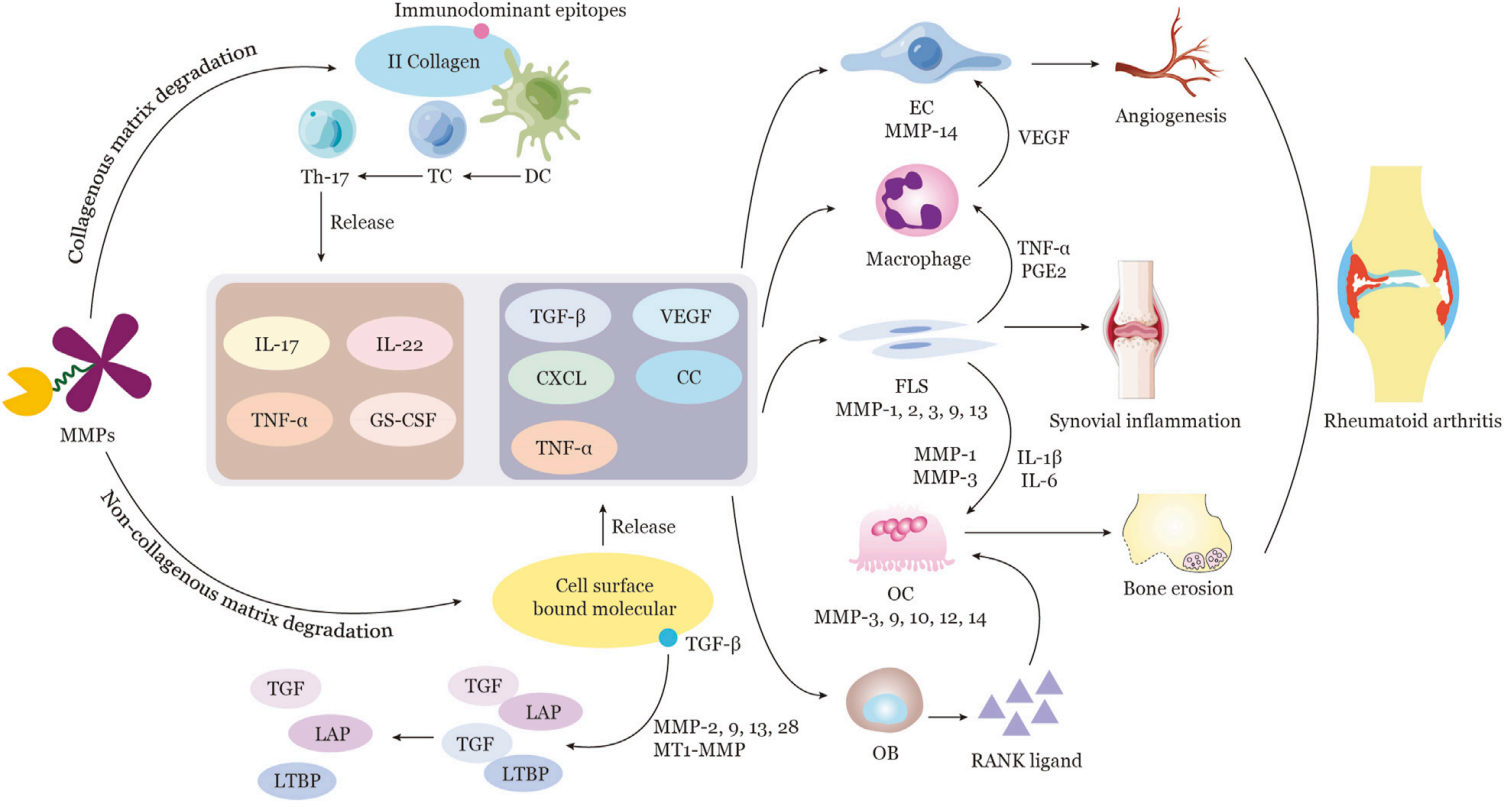
Function of MMPs in the Pathogenesis of Rheumatoid Arthritis MMPs are capable of proteolytically cleaving a variety of ECM components, including collagen and non-collagen. MMPs such as MMP1, 8, 13, and MT1-MMP are responsible for the degradation of tissue components in the joint vicinity. Furthermore, MMP9 is able to break down II collagen fragments to generate immunodominant epitopes for dendritic cell (DC) recognition and activation. Subsequently, DCs transmit the signal to T cells, leading to T-cell proliferation and polarization to Th17 cells. Activated Th17 cells then secrete a range of cytokines, including IL-17, IL-22, TNFα, and Granulocyte Colony-Stimulating Factor (GC-CSF). Certain MMPs possess the ability to cleave ECM proteins and selectively release cell-surface-bound molecules, such as cytokines, growth factors, and their receptors, which are involved in the regulation of inflammation and angiogenesis. For instance, MMP2, 9, 13, 28, and MT1-MMP are capable of directly activating TGFβ by releasing it from a complex of TGFβ-latency-associated peptide (LAP), as well as by cleaving ECM-bound latent TGFβ binding protein (LTBP) and a soluble form of LTBP. The production of various cytokines, growth factors, and other factors stimulate the activation of endothelial cells, macrophages, synovial fibroblasts, osteoblasts, and osteoclasts directly or indirectly. The resulting complex cellular interactions, mediated by multiple cytokines and MMPs released from activated cells such as ECs, FLSs, and OCs, culminate in angiogenesis, synovial inflammation, and bone erosion in RA. Note: Extracellular matrix, ECM; TGFβ-latency-associated peptide, LAP; latent TGFβ binding protein, LTBP; endothelial cell, EC; synovial fibroblast, FLS; osteoclast, OC.

In RA, multiple cells can produce MMPs, such as fibroblast-like synoviocytes (FLS), osteoclasts (OC), endothelial cells (EC), chondrocytes, and neutrophils (as displayed in Table 1; Figure 2). FLS can express almost all the MMPs exclude MMP8 and MMP20 (Konttinen et al., 1999; Tolboom et al., 2002). It has an ability of invasive properties by expressed MMP1, MMP3, or MMP10 (Tolboom et al., 2002), and can produce MT1-MMP and constitutive MMP2 and induced MMP9 to further degrade cartilage type II collagen and bone type I collagen into (auto)immunodominant epitope thus maintains the aggressive phenotype of the advancing pannus (Sabeh et al., 2010). Infiltrating neutrophils is the primary sources of MMP8,9,25 (Matsuda et al., 2003; Vandooren et al., 2013) and considered as an important cell in the RA disease (Grillet et al., 2023). It can produce MMP9 and MMP8 to activate MMP2 via reactive oxygen species or to induce cartilage collagen breakdown, angiogenesis and generate type II collagen fragments as autoantigenic remnant epitopes (Peppin and Weiss, 1986; Opdenakker et al., 2001; Ardi et al., 2007). And MMP8 could enhance neutrophil migration to the site of inflammation by cleaving CXCL6, which maintains the inflammatory status (Tester et al., 2007). EC can express MMP2,3,9,14 to involve in vascular remodeling (Lozito and Tuan, 2012; Lee et al., 2015; Kanayasu-Toyoda et al., 2016). MMP1, 3, 7, 9, and 13 are responsible for degrading the endothelial basement membrane and releasing angiogenic factors that stimulate endothelial cell activation and vascular proliferation (Inoki et al., 2002; Wang et al., 2021). Under different stimuli, chondrocytes are able to express MMP8 and MMP13 to cleave type II collagen in cartilage and result in the cartilage destruction (Yoshino et al., 2000; Charlier et al., 2019). Osteoclasts could release MMP3,9,10,12,14 to involve bone destruction (Bord et al., 1998; Hou et al., 2004; Cabral-Pacheco et al., 2020), particularly in MMP9 and 14 to cooperatively proteolyze the β-galactoside-binding lectin, galectin-3 on the cell surface (Ahmadzadeh et al., 2022; Zhu et al., 2023). Compared with OA disease, MMPs play a different role in the development of RA disease (Grillet et al., 2023). Studies have found that the levels of proMMP3, 8, and 9 are higher in the serum of RA patients than in those of OA or control persons (Tchetverikov et al., 2004). The level of MMP9 are upregulated by 67-fold in the joint synovial effusions of RA patients in comparison to OA patients (Ahrens et al., 1996). The concentration of MMP9 in the plasma was discerned to be 7-fold higher in RA patients than in healthy people (Tchetverikov et al., 2004). It has been noted that a strong correlation exists between the levels of MMP1,3,10 and the bone destruction in RA patients (Ahrens et al., 1996). Data from single-cell sequencing indicated that MMP3 generated from synovial immune cells in anti-citrullinated protein antibodies (ACPA) negative RA patients was significantly upregulated in comparison to that of ACPA positive RA patients (Wu et al., 2021), implying that MMP3 possesses a noteworthy value as a diagnostic and monitoring marker for ACPA negative RA patients (Liang et al., 2022).

**TABLE 1:** Expression of MMPs in RA disease.

Cell type	Functions and actions of MMPs	References
Fibroblast-like synoviocytes	To express multiple MMPs exclude MMP8 and MMP20; to have an ability of invasive properties by expressed MMP1, MMP3, or MMP10	17,18
	to produce MT1-MMP and constitutive MMP 2 and induced MMP 9 to further degrade cartilage type II collagen and bone type I collagen into (auto)immunodominant epitope	19
Neutrophil	to produce MMP9 and MMP8 to activate MMP2 via reactive oxygen species or to induce cartilage collagen breakdown, angiogenesis and generate type II collagen fragments as autoantigenic remnant epitopes	23,24,25
	MMP8 could enhance neutrophil migration to the site of inflammation by cleaving CXCL6	26
Endothelial cell	MMP1, 3, 7, 9, and 13 are responsible for degrading the endothelial basement membrane and releasing angiogenic factors that stimulate endothelial cell activation and vascular proliferation	30,31
Chondrocytes	to produce MMP8 and MMP13 to cleave type II collagen in cartilage	32,33
Osteoclasts	Osteoclast function is dependent on the ability of MMP9 and MMP14 to cooperatively proteolyze the β-galactoside-binding lectin, galectin-3, on the cell surface	36,37

## 4 Function of MMPs in rheumatoid arthritis

### 4.1 The role of MMPs in ECM remodeling

The ECM is a highly dynamic intercellular milieu closely associated with the cellular status. It is composed of the interstitial connective tissue matrix and a basement membrane (BM). The ECM remodeling process consists of ECM degradation and production, which regulate various cellular behaviors (Bonnans et al., 2014). In the vicinity of the joint, MMP1, 8, 13, and MT1-MMP are the major hydrolases responsible for the breakdown of tissue components, and MMP9 can furtherly degrade type II collagen fragments to generate immunodominant epitope (Vankemmelbeke et al., 1998; Van den Steen et al., 2002; Van den Steen et al., 2004) (Shown in Figure 2; Table 2). In RA, it has been reported that components of the non-collagenous matrix of the joints can be degraded by elevated MMP2,3, and 9 (Burrage et al., 2006), and degradation of aggrecan and cartilage oligomeric matrix protein (COMP) requires participation of MMP19 (Stracke et al., 2000). MMP1 and 13 appear to predominate in RA due to their capacity to restrict collagen degradation rates. Additionally, MMP13 is also capable of degrading aggrecan and proteoglycan, suggesting it has a dual role in ECM destruction in RA (Burrage et al., 2006; Takaishi et al., 2008). ECM remodeling is further linked to the balance of MMPs and endogenous TIMPs. The intricacies of the equilibrium between MMPs and TIMPs have been documented (Laronha and Caldeira, 2020). In a physiological setting, MMPs and TIMPs form a 1:1 non-selective complex to take part in ECM homeostasis (Bode and Maskos, 2003). When this balance is disrupted due to increased production of MMPs or a lack of adequate regulation by TIMPs, tissue remodeling is disregulated, which underpins the pathogenesis of RA.

**TABLE 2:** Function and action of MMPs in RA disease.

Function	MMPs	Action in RA
Extracellular matrix remodeling	MMP1/2/3/8/9/11/13/14/28	Break down the components of the collagenous and/or non-collagenous matrix of the joints; activate TGFβ,VEGF,TNFα, and other chemokine and cytokine to promote cell migration and blood vessel formation; maintain balance of MMPs and TIMPs
Angiogenesis	MMP1/2/3/7/9/13/14/19	Activate endothelial cell directly or indirectly; promote release of angiogenic factors, such as PDGF, VEGF,FGFR,EGF,TGFβ, and so on; drive the pro-inflammatory response and relative vascular hyperplasia signal pathway
Oxidative stress and nitrosative stress	MMP1/2/3/8/9/13/14	Contribute to regulate MMP activity inside the cell; increase MMP activation and take participate in the pathologic modifications of biologic functions of MMPs
Cell migration and invasion	MMP1/2/9/12/13/14/17/28	Stimulate invasive pseudopodia generation and promote destruction of the basement membrane; activate migratory signals to enhance cell migration; as a negative regulator of macrophage recruitment to anti-inflammation
Cytokine and chemokine processing	MMP1/2/3/7/8/9	As upstream of articular cartilage and bone destruction events; involve in inflammatory process, such as regulating release of TNFα and IL-1β; contribute to the modification of chemotactic agents to regulate cell chemotaxis and migration to the inflammation site; activate, inactivate or antagonize the biological functions of cytokines and chemokines
Inflammasome	MMP-3/9/13	Actively respond to the call of inflammasome and remove aging and necrotic tissues; release more DAMPs to regulate the inflammasome, leading to a vicious circle
PANoptosis	MMP2/3/9/13/15	Induce cell apoptosis by cleaving the nuclear matrix; mainly involve in angiogenesis, FLS survival, balance between osteoblast/chondrocytes and osteoclasts, and between pro-inflammatory and anti-inflammatory cell
Bone destruction	MMP1/2/3/7/9/10/13/14	Directly adheres to the surface of chondrocytes to causes cartilage damage; regulate inflammatory cytokines and chemokines release; promotes cell migration and invasive angiogenesis and initiate osteogenic and osteoclast balance signals

### 4.2 The role of MMPs in angiogenesis

**FIGURE 3:**
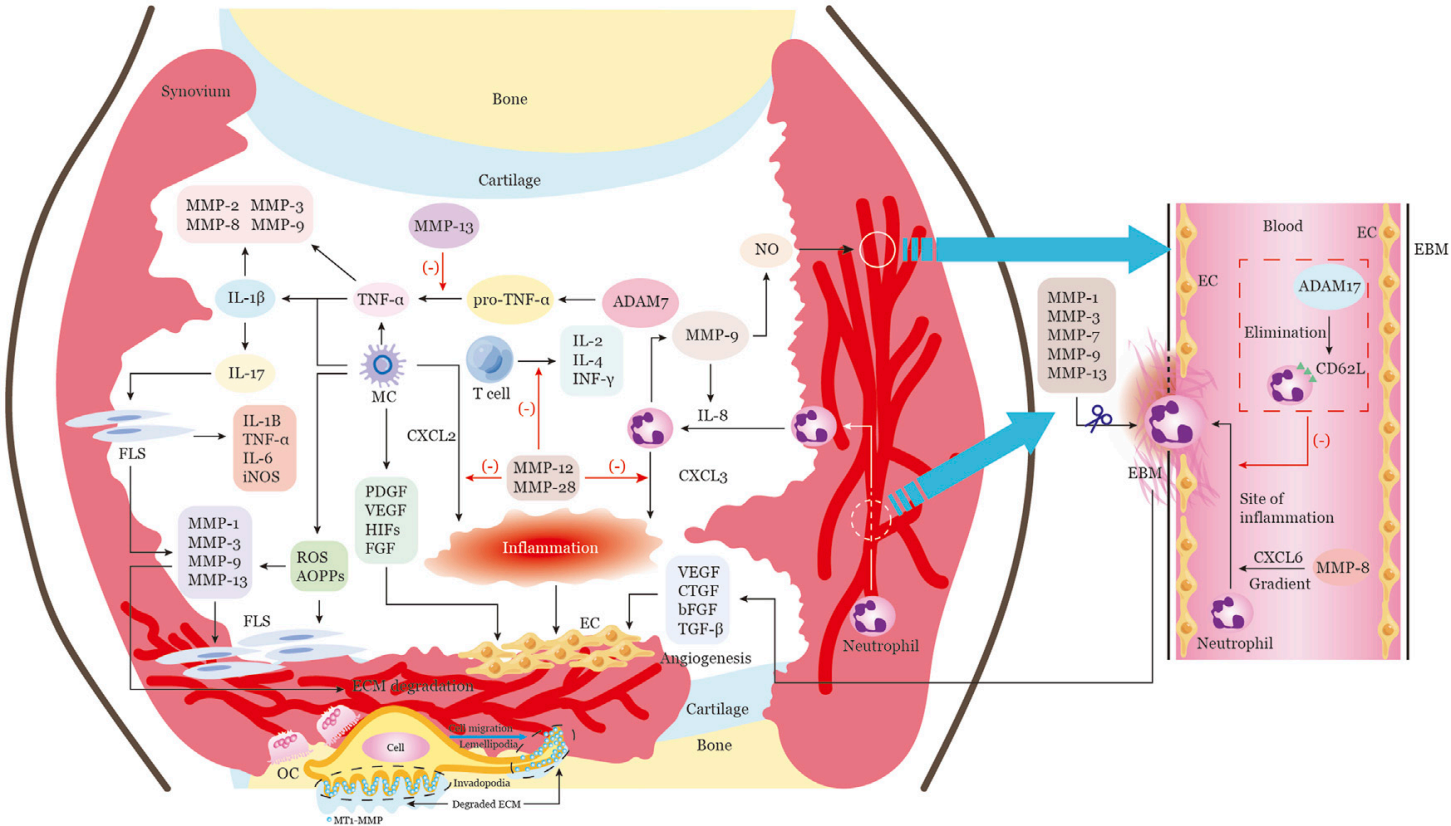
Multiple functions of MMPs in the RA articular cavity extracellular microenvironment. The progression of RA involves multiple stages, all of which can be modulated by MMPs and other extracellular proteinases. These stages include vascular proliferation, inflammatory cell infiltration, and bone erosion. Inflammatory cells from the peripheral blood or inflamed synovial tissue migrate into the joint cavity, creating an immune microenvironment that provides continuous inflammatory stimulation of synovial tissue proliferation. MMPs in RA are mainly produced by infiltrating cells such as macrophages, endothelial cells, neutrophils, and synovial fibroblasts. The proliferation and activation of endothelial cells leads to massive angiogenesis, which is an essential component of pannus. Additionally, soluble cytokines, growth factors, and chemokines are released by macrophages, neutrophils, and other cells in the synovia, which help to activate endothelial cells and attract endothelial progenitor cells. MMPs such as MMP1, 3, 7, 9, and 13 are responsible for degrading the endothelial basement membrane and releasing angiogenic factors that stimulate endothelial cell activation and vascular proliferation. MMP-8 enhances neutrophil migration to the site of inflammation by cleaving CXCL6, while ADAM17 inhibits neutrophil extravasation across the endothelial barrier by cleaving CD62L from the surface of neutrophils. MMP-9 activates IL-8, creating a positive feedback loop for neutrophil chemotaxis. Additionally, MMP-12 and MMP-28 can cleave and inactivate CXCL2 and CXCL3, respectively, reducing neutrophil and macrophage chemotaxis. MMP-12 also inhibits T-cell proliferation and function, reducing the secretion of pro-inflammatory molecules such as IL-2, IL-4, and IFNγ. Macrophages also release oxidative and nitrosative stress molecules, such as ROS, RNS, and AOPPs, which induce inflammatory responses and invasive behavior in fibroblasts, resulting in the release of MMPs. MMPs can then regulate the production of multiple cytokines, such as TNFα and IL-1β, which in turn induce the synthesis and secretion of MMPs. Interleukin-17 can activate fibroblasts to release numerous pro-inflammatory factors and MMPs, which promote extracellular matrix degradation and fibroblast proliferation. This proliferating synovial tissue then forms the pannus and climbs onto the surface of the cartilage, leading to cartilage and bone damage. Finally, active MT1-MMP, which is enriched at the invading pseudopodia-associated plasma membrane of migrating cells, promotes cell migration and invasion by degrading the extracellular matrix barrier and activating osteoclasts, further accelerating bone erosion. Note: Macrophages, MC; endothelial cells, EC; synovial fibroblast, FLS; platelet derived growth factor, PDGF; vascular endothelial growth factor, VEGF; basic fibroblast growth factor, bFGF; transforming growth factor beta,TGFβ; the CXC-motif ligands, CXCL; a disintegrin and metalloproteinases 17, ADAM17; reactive oxygen species,ROS; reactive nitrogen species, RNS; advances oxidation protein products, AOPP; inducible nitric oxide synthase, iNOS; osteoclast,OC.

Angiogenesis is a vital process in the etiology and progression of RA due to its ability to facilitate leukocyte recruitment, synovial hyperplasia, and the formation of aggressive pannus tissue (shown in Figure 3). Newly formed vascular structures are critical for the establishment and maintenance of RA pannus. Numerous studies have demonstrated the essential role of MMP2, 9, 19, and MT1-MMP in angiogenesis regulation (van Hinsbergh et al., 2006; Elhaj Mahmoud et al., 2021). These molecules contribute to vascular remodeling by modulating platelet-derived growth factor (PDGF) signaling, catalyzing the proteolysis of type I collagen, controlling perivascular smooth muscle cells, and mobilizing VEGF and other factors (Ahrens et al., 1996) (shown in Table 2). MMPs can also increase the availability and bioactivity of angiogenic factors. Studies has proved that MMP1, 3, 7, 9, 14, 16, 19 can cleave and regulate VEGF bioavailability and vascularity (Page-McCaw et al., 2007), while MMP9 holds the ability to transition the condition from vascular quiescence to active angiogenesis. Conversely, MMPs may also be able to inhibit angiogenesis by promoting the production of endogenous angiogenesis inhibitors through the proteolysis of certain collagen chains and plasminogen. For instance, MMP7, MMP9, and MMP12 are known to transform plasminogen into angiostatin, a potent angiogenesis inhibitor (Wolf et al., 2017). MMP12’s capacity to increase neutrophil infiltration and enhance epithelial cell migration (Wolf et al., 2017), suggesting that MMP12 is a multifunctional molecule. Additionally, the TGF-co-receptor endoglin can be broken down by MMP14 to counteract its angiogenic effect (Hawinkels et al., 2010). In conclusion, the precise regulation of several angiogenic agents and signaling pathways is necessary for angiogenesis.

### 4.3 The role of MMPs in oxidative and nitrosative stress

It is widely acknowledged that oxidative processes and nitrosative stress are implicated in a number of facets of RA onset and progression, such as inflammation, proliferative synovitis, muscle weakness, and bone destruction (Khojah et al., 2016; Yamada et al., 2017). Reactive oxygen and nitrogen species have been identified in the knee tissue or joint fluid of RA sufferers, which can be employed to gauge the initial response to RA treatment (Costa et al., 2018; Smallwood et al., 2018). It has been reported that the generation of reactive oxygen and nitrogen moieties can influence MMP activity within the cell (Cauwe and Opdenakker, 2010; Bassiouni et al., 2021). MMPs can be activated by ROS oxidizing their thiol group within the cysteine switch, even in the presence of propeptide (Okamoto et al., 2001; Viappiani et al., 2009). It has been demonstrated that MMP1, 2, 8, and 9 can be activated by ONOO-glutathionylation cysteinyl S-glutathiolation or Snitrosylation of cysteines 60 and 102, resulting in a disulfide S-oxide bond (Okamoto et al., 2001; Viappiani et al., 2009; Jacob-Ferreira et al., 2013). The Nuclear factor erythroid 2-related factor 2 - Kelch ECH associating protein 1 (Nrf2-Keap1) signaling pathway, an endogenous antioxidant system, has been revealed to present a protective effect in RA (Kaur et al., 2021). *In vitro* primary culture studies have demonstrated that augmented Nrf2 expression can reduce the expression of MMP2 and 9, thus inhibiting cell invasion (Kapoor et al., 2019). Advances oxidation protein products (AOPPs), a novel biomarker of oxidative stress, have been known to induce inflammatory responses and invasive behavior of FLS cells, which in turn promotes the release of MMP3 and 13 via the RAGE-NF-Κb pathway (Lou et al., 2021). Furthermore, the interaction between NO and MMP9 has been demonstrated to play a crucial role in angiogenesis (O'Sullivan et al., 2014). VEGF-stimulated MMP9 activity has been shown to mediate NO synthase isoform and focal angiogenesis (Hawinkels et al., 2008; Lee et al., 2009). Collectively, it is evident that oxidative and nitrosative stresses can promote MMP activation and are associated with the pathological alteration of MMPs biological functions, which in turn are involved in the pathophysiology of RA (shown in Table 2).

### 4.4 The role of MMPs in cell migration and invasion

The destruction of the BM of synovial cells can enable them to migrate through the tissue and permeate interior of the joint. Invadopodia, a conserved subcellular structure conformation resulting from localized ECM remodeling (Jacob and Prekeris, 2015), has been demonstrated to be implicated in BM degradation (Lohmer et al., 2014). The capacity of invadopodia to degrade the ECM is attributed to the presence of MMPs (shown in Figure 3; Table 2). It has been reported that MMP2 and MT1-MMP are essential for instigating invadopodia generation (Maybee et al., 2022). Active MT1-MMP at the plasma membrane of migrating cells could promote cell migration and invasion by directly degrading the ECM barrier to form a passageway (Itoh, 2006). MT1-MMP can also activate proMMP-2 or proMMP-13 by interacting with TIMP-2 bound to MT1-MMP to form a ternary complex or by plasmin cleaving the Lys38-Glu39 and Arg76-Cys77 peptide bonds in the propeptide domain in order to degrade the ECM barrier indirectly, respectively (Knauper et al., 1996; Nagase, 1998). MT1-MMP can also shed cell surface molecules, such as cluster of differentiation-44 (CD44) and syndecan 1, activate migratory signals ERK, and release ECM fragments laminin 5 to enhance cell migration (Itoh, 2006). Additionally, the plasma membrane delivery of MT1-MMP, as well as the secretion of MMP2 and MMP9, are closely related to the level of cortactin expression, which is the fundamental element and a particular marker of invadopodia (Clark et al., 2007). It has been shown that cortactin can regulate MT1-MMP expression on the cell surface. Thus, the consolidated activity of MMP2, MMP9, and MT1-MMP is considered to be the critical step in initiating the degradation of localized BM during synovial proliferation (Chen and Wang, 1999). MT4-MMP can also induce invadopodia formation by binding with Src substrate Tks5 and platelet-derived growth factor receptor alpha (PDGFRα), which results in Src activation and promotes amoeboid-like movement (Yan et al., 2020). MMP28 is an another important MMP in cell migration and invasion. But differs from the majority of other MMPs, MMP28 expresses in numerous normal tissues and plays an indispensable role in sustaining tissue homeostasis (Knauper et al., 1996). Although evidence from RA is still scarce, data from Mmp28−/− mice shows that macrophages can migrate faster and release more macrophage inflammatory protein such as MIP-1α and MIP-1β, as well as elevated MMP9(79), which suggesting the important role of MMP28 in reducing cell migration and preserving tissue homeostasis in inflammatory disease.

### 4.5 The role of MMPs in cytokine and chemokine processing

The increased expression of MMPs has been associated with the involvement of cytokines in the inflammatory process in RA (Shown in Figure 3; Table 2). TNFα has been found to induce the expression of MMP2, 3, 8, and 9 through a cascade signaling pathway, such as mitogen-activated protein kinase (MAPK), protein kinase C (PKC), protein kinase D1 (PKD1), mitogen-activated protein/extracellular signal-regulated kinase (MEK), and protein kinase B (AKT) (Chen and Wang, 1999; Yan et al., 2020). The synthesis of TNFα as a membrane-anchored precursor (pro-TNFα) requires protease and metalloproteinase domain-containing protein 17 (ADAM17) to cleave and release its extracellular domain of soluble 17-kDa (Bister et al., 2004). IL-1β has been shown to activate MMP2, 3, and 9, but the proteolytic activity of these MMPs can inhibit IL-1β activation, suggesting an important role of MMPs in respect of keeping level of IL-1β (Yoo et al., 2011; Ma et al., 2012). IL-17 has been demonstrated to increase the expression of MMP1 and 3 on myofibroblasts, and this effect was further amplified when combined with TNFα and IL-1β (Du et al., 2019). Chemokines have also been identified as direct targets of MMPs that can inhibit the chemotactic attractants of these molecules (Horiuchi et al., 2007). It is very important for efficient neutrophil migration to sites of infection by chemotactic gradients and extravasation through blood vessels and tissues (Khokha et al., 2013). Studies have shown that MMP9 can increase the potency of IL-8 (functionally analogous to CXCL6 in mice) by a factor of ten (Van den Steen et al., 2000), while MMP2 can render the mononuclear cell-attracting chemokine CCL7 (also known as monocyte chemoattractant protein-3; MCP-3) inactive (McQuibban et al., 2000). Animal experiments have confirmed that MMP7 deficiency can compromise the CXCL3 chemokine gradient (Li et al., 2002) and neutrophils failed to migrate to sites of lipopolysaccharide (LPS) administration in Mmp8−/− mice (Tester et al., 2007). MMP2 and MMP9 have been reported to recruit immune cells by forming a chemokine gradient of both the CC- (e.g., CCL7) and the CXC-motif (e.g., CXCL12, CXCL6, and CXCL8) ligands (Overall, 2002). MMP2 can clean CCL7 and bind to CC-chemokine receptor (CCR) 1, 2, and 3 (McQuibban et al., 2000), while MMP9 can activate CXCL6 and CXCL8 and inactivate CXCL1 and 4 (Van den Steen et al., 2000). Taken together, MMPs have the ability to activate, inactivate, or antagonize cytokines and chemokines by proteolytic processes, which can either promote or suppress inflammation (Nissinen and Kahari, 2014).

### 4.6 The role of MMPs in PANoptosis

PANoptosis has been determined to be an inflammatory programmed cell death (PCD) pathway that is distinct from pyroptosis, apoptosis, and/or necroptosis, exhibiting key characteristics of each. Reports have indicated that it has a close correlation with arthritis (Wang and Kanneganti, 2021). It has been demonstrated that a deficiency in the deubiquitinating enzyme A20 can lead to inflammasome activation, in turn causing RIPK1 kinase-dependent, RIPK3-MLKL-mediated necroptosis and inflammatory arthritis in mice (Polykratis et al., 2019). Additionally, a missense mutation in pstpinp2 has been shown to result in bone disruption, implicating it in the development of an autoinflammatory disorder (Cassel et al., 2014; Lukens et al., 2014). Although evidence of PANoptosis directly relating to RA disease is scarce, various studies have demonstrated that PANoptosome is strongly associated with RA, particularly NLRP3 inflammasome. It has been established that NLRP3-mediated pyroptosis plays a pivotal role in the progression of RA, releasing increased amounts of IL-1β and IL-18, which, in turn, expedite the advancement of the disease (Chen et al., 2021). Additionally, augmented TNFα and IFN-γ concentrations in the circulation are associated with a poorer prognosis in RA patients (Azizieh et al., 2017), and they have been observed to induce PANopotosis synergistically by way of the JAK/STAT1/IRF1 signaling pathway and NO production (Karki et al., 2021). There has been a focus on MMPs in terms of their regulation of cell apoptosis and proliferation (Mannello et al., 2005a). It is known that FLS are insensitive to apoptosis, which, together with an imbalanced regulation of cell apoptosis in osteoblast/chondrocytes, osteoclasts and decreased apoptosis of proinflammatory cells, can lead to RA progression (Zhao et al., 2021). Consequently, targeting MMPs to induce proinflammatory cells apoptosis and maintain the balance in osteoblast/chondrocytes, osteoclasts in RA may serve as a promising treatment approach.

### 4.7 The role of MMPs in bone destruction

MMPs have been implicated in the bone destruction associated with RA, primarily through three mechanisms: 1) Adhesion to chondrocytes, leading to degradation of collagen and consequent cartilage damage; 2) Regulation of inflammatory cytokines and chemokines, resulting in an imbalance of homeostasis in the affected joint and activation of inflammatory signaling pathways that promote osteoclast differentiation and bone resorption; 3) Promotion of cell migration and invasive angiogenesis, initiating osteoblast and osteoclast balance signals and accelerating bone destruction (details showed in Figure 3). In the early stages of RA, fibrosis and lack of proteoglycans is observed on the cartilage surface, even in the absence of pannus tissue (Cabral-Pacheco et al., 2020). Under different stimuli, chondrocytes are able to express various proteases, including MMP1, 2, 3, 7, 8, 9, 10, 13, 14, ADAM9, 10, 17, ADAMTS4, and other types initiating cartilage destruction directly. Evidence suggests that FLS are involved in both synovial inflammation and bone erosion in RA, through the production of various factors such as IL-1β, TNFα, IL-6, IL-8, MMP1, and MMP13 (Wang et al., 2002; Mu et al., 2016). IL-1β is capable of inducing a variety of MMPs, including MMP1, 3, 8, 13, 14, 29 and activating osteoclasts to break down the cartilaginous matrix (Amarasekara et al., 2018). MMP13 activity is essential for cartilage erosion (Ericsson et al., 2019). The activation of MMPs is essential for cell migration, as the ECM must be rearranged to permit cellular movement (Little et al., 2009). MMP12 has been identified as playing an invasive role in the macrophages’ penetration of reconstituted basement membrane (Shipley et al., 1996).

## 5 MMPs and drug in rheumatoid arthritis

There are some challenges in the current management of RA. Approximately 30%–40% of all biological agents have a limited effect on inhibiting bone destruction (Svenson et al., 2007). MMPs inhibitors have been tested in numerous rodent models of inflammation. As shown in Tables 3, 4 , we summarized the details of MMPs inhibitor in pre-clinical study (updated according to the Selleck’s official website:https://www.selleck.cn/mmps.html) and clinical trials of MMPs related to inflammatory and immune diseases. FR255031, a novel synthetic MMPs inhibitor, was observed to significantly reduce cartilage degradation in a dose-dependent manner and decrease the levels of collagenases (MMP1, MMP8 and MMP13), gelatinases (MMP2 and MMP9) and membrane type 1 MMP (MT1-MMP/MMP14) in the CIA model (Ishikawa et al., 2005). Butanediamide, as a potent MMPs inhibitor, showed significant remission in edema, pannus formation, new bone growth periosteum, and bone marrow osteoclasts in a rat model of chronic destructive arthritis induced by adjuvant (Conway et al., 1995). GW3333, a dual inhibitor of TNFα converting enzyme (TACE) and MMPs, exhibited inhibition of both ankle swelling and joint destruction, whereas an anti-TNFα antibody had no effect when used alone (Conway et al., 2001). JNJ0966 and MMP-9-IN-1 are both highly selective compounds targeted MMP9. But they work through completely different mechanisms. JNJ0966 can inhibit the activation of MMP9 zymogen and the subsequent generation of catalytically active enzymes (Scannevin et al., 2017), while MMP-9-IN-1 binds to the hemopexin (PEX) domain of MMP-9 resulting in the abrogation of MMP-9 homodimerization and blockage of a downstream signaling pathway required for MMP-9-mediated cell migration (Dufour et al., 2011). CL-82198 is a highly selective MMP13 inhibitor (Paramakrishnan et al., 2023), which has been used to treat Alzheimer’s disease (Paramakrishnan et al., 2023), liver fibrosis (George et al., 2017), chronic periodontitis (Hernandez Rios et al., 2009). However, the effect of these inhibitors on human arthritis is still in need of further investigation. Batimastat (BB-94) (Taraboletti et al., 1995; Botos et al., 1996), marimastat (BB-2516) and ilomastat (GM6001) (Leppert et al., 1995; Li et al., 2002) are structurally similar MMP inhibitor, belong to hydroxamate-based inhibitors, which are capable of inhibiting MMPs expression, and have been in use for many years. However, their clinical performance was ultimately found to be unsatisfactory due to their extensive inhibitory effect on many MMPs (Close, 2001). SB-3CT(Bonfil et al., 2006), NSC 405020(Remacle et al., 2012), T-26c (Nara et al., 2014) and ARP 100(Rossello et al., 2004) are four new promising MMP inhibitor, which has no reports related to arthritis. Cipemastat (also known as Ro32-3555), an inhibitor of MMP1, MMP3 and MMP9 used for the therapy of RA and OA, failed to show any reduction in the process of joint damage in patients with RA in a clinical trial (Milner and Cawston, 2005). Doxycycline, an antibiotic known to weakly inhibit MMPs, and some of its derivatives have been demonstrated to have an effect on inhibiting MMPs (Milner and Cawston, 2005). Nevertheless, a long-term observational study in the USA indicated that patients initiating doxycycline had higher disease activity, more comorbidities, and more prior use nonbiologic disease-modifying anti-rheumatic drugs (DMARDs). Additionally, side effects were reported by 11.8% of doxycycline users (Smith et al., 2011). Andecaliximab (also known as GS-5745), a highly selective monoclonal antibody against MMP9, is the only drug targeting MMPs that has been used in RA clinical trials. In a double-blind phase 1b trial on andecaliximab, the average MMP9 coverage was maintained at 80% after the initial administration of andecaliximab, indicating good drug tolerance. The most frequent adverse event was allergy (13.3%) (Gossage et al., 2018). Nevertheless, there is still insufficient relevant research on MMP9 in RA progression, bone and joint damage. Additionally, many phytochemicals targeting MMPs have good effects on regulation tissue degradation in inflammation and RA, such as flavonoids, glycosides alkaloids, lignans and terpenes (Alamgeer et al., 2020). Taken together, clinical use of MMP inhibitors requires firm proof of MMPs and knowledge about the cells and molecules involved in RA entities (Grillet et al., 2023).

**TABLE 3:** Pre-clinical study of MMPs inhibitor.

Name	Targets	Effects	Models	Dosages	Administration	References
FR255031	MMP1, MMP2,MMP8,MMP9, MMP13,MMP14	significantly reduce cartilage degradation, bone destruction and pannus formation in a dose-dependent manner by decreasing the levels of collagenases, gelatinases and membrane type 1 MMP.	CIA rat	100 mg/kg/d	Orally	105
Butanediamide	MMPs	a direct role for MMP in cartilage and bone damage and pannus formation	AIA-rats	6, 12, and 25 mg/kg/d	osmotic minipumps	106
GW3333	TACE and MMPs	inhibit TNF-alpha converting enzyme and MMPs	CIA rat	80 mg/mg/d	Orally	107
JNJ0966	MMP9	a highly selective compound that inhibits the activation of MMP9 zymogen and the subsequent generation of catalytically active enzymes	—	10 μM	—	108
MMP-9-IN-1	MMP9	a specific inhibitor of MMP-9, selectively binds to the hemopexin (PEX) domain of MMP-9 resulting in the abrogation of MMP-9 homodimerization and blockage of a downstream signaling pathway required for MMP-9-mediated cell migration	cancer cell lines	2.1 μM	—	109
CL-82198	MMP13	a selective and orally MMP13 inhibitor	zebrafish	10 μM	—	110
Batimastat (BB-94)	MMP1, MMP2, MMP9, MMP7 and MMP3	exhibits an unexpected binding geometry with the thiophene ring deeply inserted into the primary specificity site, pointing the way for the design of a new generation of potential antitumor drugs	Tumor cell	—	—	113
		reduces *in vivo* growth of experimental hemangiomas by blocking endothelial cell recruitment by the transformed cells or by interfering with cell organization in vascular structures	Mouse	30 mg/ml/d	Intraperitoneal injection	114
Ilomastat (GM6001)	MMP1, MMP2, MMP3, MMP7, MMP8, MMP9, MMP12, MMP14, and MMP26	to inhibit T cell homing by interference with production of gelatinase from T cells	Human T cells	—	—	115
		to inhibit intimal hyperplasia and intimal collagen content, and increase lumen area in stented arteries without effects on proliferation rates	Rabbit	100 mg/kg/day	Subcutaneous injection	116
SB-3CT	MMP2 and MMP9	directly inhibit bone marrow endothelial cell invasion and tubule formation	mouse	50 mg/kg/day	Intraperitonea	118
NSC 405020	MMP14	It is an MT1-MMP inhibitor that directly interacts with the PEX region of MT1-MMP, affecting PEX dimerization but not MT1-MMP catalytic activity	BALB/c nu/nu mice	0.5 mg/kg, three times a week	Intratumoral injection	119
T-26c	MMP13	a potent and selective inhibitor of MMP13, which is 2,600 times more selective than other related metalloproteinases	—	6.9 p.m.	—	120
ARP 100	MMP2	a biphenylsulfonamide MMP inhibitor which shows selectivity towards MMP2	rat hearts	10 μM	—	121

**TABLE 4:** Clinical trials of MMPs related to inflammatory and immune disease.

Name	Targets	Clinical Trials.gov identifier	Condition or disease	Phase
Andecaliximab	MMP9	NCT03631836	Recurrent Glioblastoma	Phase 1
		NCT02759562	Adults With Cystic Fibrosis	Phase 2
		NCT02862535	Gastric Adenocarcinoma	Phase 1
		NCT02864381	Gastric Adenocarcinoma	Phase 2
			Gastroesophageal Junction Adenocarcinoma	
		NCT02545504	Gastric Adenocarcinoma	Phase 3
			Gastroesophageal Junction Adenocarcinoma	
		NCT02520284	Ulcerative Colitis	Phase 2
				Phase 3
		NCT02405442	Crohn’s Disease	Phase 2
		NCT02176876	Rheumatoid Arthritis	Phase 1
		NCT02209987	Healthy Adults	Phase 1
		NCT02862574	Rheumatoid Arthritis	Phase 2
		NCT02077465	Chronic Obstructive Pulmonary Disease	Phase 1
		NCT01803282	Advanced Solid Tumors	Phase 1
		NCT01831427	Ulcerative Colitis	Phase 1
FP-025	MMP12	NCT03304964	Healthy Adults	Phase 1
		NCT03858686	Asthma, COPD	Phase 2
		NCT02238834	Healthy Adults	Phase 1
		NCT04750278	Severe to Critical COVID-19 With Associated ARDS	Phase 2
				Phase 3
PG-530742	MMP	NCT00041756	Osteoarthritis, Knee	Phase 2
		NCT00067236	Myocardial Infarction	Phase 2
			Heart Failure	
			Heart Enlargement	
Marimastat	MMP1,MMP2,MMP7, MMP9 and MMP14	NCT00002911	non-small cell lung cancer	Phase 3
		NCT00003011	Small Cell Lung Cancer	Phase 3
		NCT00003010	breast cancer	Phase 3
ARO-MMP7	MMP7	NCT05537025	Idiopathic Pulmonary Fibrosis	Phase 2
BT1718	MT1-MMP	NCT03486730	Advanced Solid Tumours	Phase 1
				Phase 2
FCX-013	MMP1	NCT03740724	Morphea	Phase 1
			Scleroderma, Localized	Phase 2
			Scleroderma	
Triolein	MMP1	NCT00004418	Male Children With Adrenoleukodystrophy	Phase 2

## 6 Conclusion

The initial hypothesis concerning MMPs inhibition was that it would impede ECM remodeling, thus inhibiting cell invasion and cancer metastasis by broadly impeding the proteolytic activity of the molecules (Mohammed et al., 2003). Recent research has revealed that distinct types of MMPs are capable of functioning as upstream molecules of signaling cascades, modulating the differential expression of molecules during ECM remodeling. Consequently, it is now evident that MMPs are not simply downstream components receiving various signals, but can also act as upstream signals that regulate multiple biological activities. This review acknowledges the role of MMPs as a pleiotropic group of molecules that are involved in several facets of RA pathogenesis, encompassing ECM remodeling, angiogenesis, cell migration and invasion, oxidative and nitrosative stress, PANoptosis, cytokine and chemokine processing, and bone destruction. Furthermore, MMPs have been demonstrated to be implicated in the pathophysiology and pharmacology of RA, with significant over-expression of MMP1, MMP3, MMP7, MMP8, MMP9, MMP12, MMP13, MMP19, and MMP25, and a concurrent downregulation of MMP17, MMP24, and MMP28. Additionally, serum and SF levels of MMP3 and MMP9 may serve as biomarkers of active RA.

However, there are still many unknowns in the usage of MMPs inhibitors to treat RA. MMPs degrade the surrounding tissue, which can be prevented by a selective MMP inhibitor to avert further tissue destruction, such as bone and joint damage, but it is unable to restore already damaged tissue, leading to chronic inflammation associated with RA. Therefore, a MMP inhibitor with potential clinical applicability should be stage-specific (Mannello et al., 2005b; Overall and Kleifeld, 2006). As MMPs are part of a check-and-balance system, the optimal combination of four TIMPs and multiple MMPs requires a 1:1 ratio. Thus, one should consider the potential adverse effects of blocking MMPs, as it can have pro-inflammatory or anti-inflammatory effects, pro-angiogenic or anti-angiogenic effects, pro-migratory or anti-migratory effects, as well as pro-apoptotic or anti-apoptotic effects. Thus, before using MMP inhibitors, it is essential to measure the levels of MMPs depending on the disease stage in RA patients. Single MMP inhibitors can be a double-edged sword, and thus additional preclinical research is needed before it is translated into clinical practice. Precision medicine targeting MMPs in RA has arrived.
